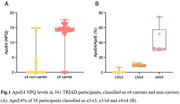# Determining APOE ε4 carriership by NUcleic acid Linked Immuno‐Sandwich Assay (NULISA)

**DOI:** 10.1002/alz.091535

**Published:** 2025-01-09

**Authors:** Kübra TAN, Andrea L. Benedet, Bingqing Zhang, Nesrine Rahmouni, Wagner Scheeren Brum, Ilaria Pola, Guglielmo Di Molfetta, Kaj Blennow, Nicholas J. Ashton, Henrik Zetterberg

**Affiliations:** ^1^ Department of Psychiatry and Neurochemistry, Institute of Neuroscience and Physiology, The Sahlgrenska Academy, University of Gothenburg, Mölndal Sweden; ^2^ Department of Psychiatry and Neurochemistry, Institute of Neuroscience and Physiology, The Sahlgrenska Academy, University of Gothenburg, Mölndal, Gothenburg Sweden; ^3^ Alamar Biosciences, Fremont, CA USA; ^4^ McGill University Research Centre for Studies in Aging, Montreal, QC Canada; ^5^ Translational Neuroimaging Laboratory, The McGill University Research Centre for Studies in Aging, Montreal, QC Canada; ^6^ Graduate Program in Biological Sciences: Biochemistry, Universidade Federal do Rio Grande do Sul (UFRGS), Porto Alegre Brazil; ^7^ Clinical Neurochemistry Laboratory, Sahlgrenska University Hospital, Mölndal Sweden; ^8^ Centre for Age‐Related Medicine, Stavanger University Hospital, Stavanger Norway; ^9^ NIHR Biomedical Research Centre for Mental Health & Biomedical Research Unit for Dementia at South London & Maudsley NHS Foundation, London UK; ^10^ King’s College London, Institute of Psychiatry, Psychology & Neuroscience, Maurice Wohl Clinical Neuroscience Institute, London UK; ^11^ Department of Molecular Neuroscience, UCL Institute of Neurology, London UK; ^12^ UK Dementia Research Institute, University College London, London UK

## Abstract

**Background:**

The APOE ε4 variant is the largest known genetic risk factor for late‐onset sporadic Alzheimer's disease (AD). Recent blood biomarker models include APOE ε4 status with plasma p‐tau217 for higher accuracy for AD pathology. Thus, protein assays that can accurately determine ε4 carriership simultaneously with plasma p‐tau217 would be advantageous for clinical use. This study aims to evaluate the concordance between the NULISA ApoE4 protein assay and conventional genetic testing for APOE measurement.

**Method:**

We included two independent cohorts; cohort 1 (Translational Biomarker for Aging and Dementia Cohort [TRIAD]) consisted of 341 participants (mean [SD] age, 64.8[16.1] years; 213 females[62.4%]) and cohort 2 consisted of 38 participants (72.1[16.1] years; 21 females[55.2%]). APOE genotyping was determined by the TaqMan® SNP Genotyping Assay. In cohort 1, ApoE4 and p‐tau217 levels (NPQ) were quantified by the NULISAseq CNS disease panel from Alamar Biosciences. In cohort 2, ApoE4% (ApoE4/ApoE^total^) were quantified by a prototype singleplex assay from Alamar Biosciences

**Result:**

In cohort 1, we included 341 participants with APOE genotyping (ε4 non‐carriers=221; ε4 carriers=120). ApoE4 NPQ values correctly identified 88.3% of carriers and 94.1% of non‐carriers (Figure 1A). Homozygous ε2 and ε4 carriers were entirely identified. Higher ApoE4 NPQ levels were observed in ε4/ε4 carriers (median [SD], 16.4[0.52]) compared to ε3/ε4 carriers (12.3[5.11]; P<0.0001) but an overlap remained. In these participants, the simultaneously measurement of NULISA plasma p‐tau217 was significantly increased in Aβ+ participants compared to Aβ‐ participants (AUC=0.941; P<0.0001). In cohort 2, we included 38 participants with APOE genotyping (ε3/ε3, n=26; ε3/ε4, n=9; ε4/ε4, n=6) and calculated the ApoE4% from a prototype singleplex NULISA method. Here, ε3/ε3 (0.03 [1.43]), ε3/ε4 (9.37 [1.61] and ε4/ε4 (41.9 [18.0]) individuals were classified with 100% accuracy (Figure 1B).

**Conclusion:**

Our study assessed ApoE protein assays to determine APOE genotype. The ApoE4 assay within the NULISAseq CNS panel demonstrated high accuracy in distinguishing APOE ε4 carriers from non‐carriers, with some discordance. The reason for the discordance subject to further studies. However, ApoE4% quantification using a prototype singleplex assay distinguished ε4 homozygous, ε4 heterozygous, and non‐carriers with 100% accuracy. This pilot study demonstrates the ability to concurrently determine APOE status and p‐tau217 levels for more accurate diagnostic models of AD pathology.